# Identification and characterization of potential NBS-encoding resistance genes and induction kinetics of a putative candidate gene associated with downy mildew resistance in *Cucumis*

**DOI:** 10.1186/1471-2229-10-186

**Published:** 2010-08-23

**Authors:** Hongjian Wan, Zhenguo Zhao, Ahmed Abbas Malik, Chuntao Qian, Jinfeng Chen

**Affiliations:** 1 State Key Laboratory of Crop Genetics and Germplasm Enhancement, Nanjing Agricultural University, Nanjing 210095, China

## Abstract

**Background:**

Due to the variation and mutation of the races of *Pseudoperonospora cubensis*, downy mildew has in recent years become the most devastating leaf disease of cucumber worldwide. Novel resistance to downy mildew has been identified in the wild *Cucumis *species, *C. hystrix *Chakr. After the successful hybridization between *C. hystrix *and cultivated cucumber (*C. sativus *L.), an introgression line (IL5211S) was identified as highly resistant to downy mildew. Nucleotide-binding site and leucine-rich repeat (NBS-LRR) genes are the largest class of disease resistance genes cloned from plant with highly conserved domains, which can be used to facilitate the isolation of candidate genes associated with downy mildew resistance in IL5211S.

**Results:**

Degenerate primers that were designed based on the conserved motifs in the NBS domain of resistance (R) proteins were used to isolate NBS-type sequences from IL5211S. A total of 28 sequences were identified and named as cucumber (*C. sativus *= CS) resistance gene analogs as CSRGAs. Polygenetic analyses separated these sequences into four different classes. Quantitative real-time polymerase chain reaction (qRT-PCR) analysis showed that these CSRGAs expressed at different levels in leaves, roots, and stems. In addition, introgression from *C. hystrix *induced expression of the partial CSRGAs in cultivated cucumber, especially CSRGA23, increased four-fold when compared to the backcross parent CC3. Furthermore, the expression of CSRGA23 under *P. cubensis *infection and abiotic stresses was also analyzed at different time points. Results showed that the *P. cubensis *treatment and four tested abiotic stimuli, MeJA, SA, ABA, and H_2_O_2, _triggered a significant induction of CSRGA23 within 72 h of inoculation. The results indicate that CSRGA23 may play a critical role in protecting cucumber against *P. cubensis *through a signaling the pathway triggered by these molecules.

**Conclusions:**

Four classes of NBS-type RGAs were successfully isolated from IL5211S, and the possible involvement of CSRGA23 in the active defense response to *P. cubensis *was demonstrated. These results will contribute to develop analog-based markers related to downy mildew resistance gene and elucidate the molecular mechanisms causing resistance in IL5211S in the future.

## Background

Recently, genes that confer resistance (R) to different types of pathogens, including viruses, bacteria, fungi, and nematodes, have been cloned by map-based cloning and transposon tagging procedures [1-4]. Apart from the *Hm1 *and *Mlo *genes [5,6], amino acid sequence comparison analyses from cloned R genes revealed that they are highly structurally conserved, such as the previously reported conserved domains leucine zipper (LZ), NBS, LRR, transmembrane (TM), and serine/threonine protein kinases (PKs) [1,7]. Of these cloned R genes, the largest group consists of the NBS-LRR family, which is characterized by an N-terminal NBS and C-terminal LRRs. Based on the presence or absence of a toll and interleukin-1 receptor (TIR) domain at the N-terminus of plant NBS-LRR R genes, they were divided into two subclasses: (1) TIR-NBS-LRR, and; (2) non-TIR-NBS-LRR type [8]. In addition, the last residue of the kinase-2 motif of identified NBS-LRR R genes, D (Aspartate) or W (Tryptophan), has also been used to predict (95% accuracy) whether they belong to the TIR or non-TIR subclass of NBS-LRR R genes [8].

Eight conserved motifs have been identified in the NBS domains of known NBS-LRR R genes [8]. Some are specific to the non-TIR or the TIR-NBS-LRR subfamily, such as RNBS-A-TIR and RNBS-D-TIR in the TIR subclass, and RNBS-A-nonTIR and RNBS-D-nonTIR in the nonTIR subclass [8,9]. Other conserved motifs such as P-loop (kinase-1a), kinase-2, kinase-3a, and GLPL are widely present in both classes. Conserved motifs in such resistance genes in different plants offer a way to isolate RGAs related to other resistance genes. Currently, RGAs isolated using this approach have been obtained extensively by PCR amplification, with degenerate primers designed based on the conserved domain of NBS-LRR, such as potatoes [10], soybeans [11], lettuce [12], barley [13], coffee [14], sunflower [15], strawberry [16], and ginger [17]. Many cloned RGAs are either closely linked to known R gene loci or are arranged in clusters similar to R genes. Consequently, degenerate RCR could be a promising approach that may facilitate the isolation of resistance genes.

Downy mildew [*Pseudoperonospora cubensis *(Berk. & M.A. Curtis) Rostovzev] is a disease that infects cucumber (*Cucumis sativus *L.) worldwide, where it can be devastating to European [18] and North American growers [19]. In China, it can cause yield loss of up to 10~30% in a regular year, or more than 80% during an epidemic year [20]. Recently, a downy mildew epidemic infected cucumber plants in North Carolina and the Delmarva Peninsula of New Jersey. The epidemic started in Florida and found its way to locations as far away as Michigan, Ohio, and then Ontario, Canada. The outbreak was evidence that some of the previously identified resistance genes (*dm-1*, *dm-2*, and *dm-3*) did not provide adequate disease control [21]. Further, several fungicides that should have provided control were ineffective because of the new pathogenicity of *P. cubensis*. Therefore, improving the genetic resistance of cucumber to downy mildew through plant breeding could be an effective way to control this disease. The narrow genetic base of modern cucumber cultivars and few sources of resistance make downy mildew resistance an important objective in cucumber-breeding programs. Therefore, the identification of new sources of resistance to downy mildew is desirable [21].

High resistance exists in wild *Cucumis *species [22-24]. Since 1971, major efforts to create interspecific hybrids by introgression of alien disease resistant genes from exotic germplasm into elite lines have been unsuccessful [25]. However, we have carried out a successful interspecific hybridization between *C. hystrix *Chakr. (a wild species with 24 chromosomes found in China) and cucumber (2n = 14) [26]. In previous studies, extensive deoxyribonucleic acid (DNA) introgression from *C. hystrix *in the progenies from the subsequent backcrossing of interspecific hybrid species (*C*. × *hytivus*, amphidiploid, 2n = 38, maternal parent) to *C. sativus *was observed [27-29]. Of the germplasms developed by alien introgression, line IL5211S (BC_1_F_7, _See "Materials and Methods") with downy mildew resistance was identified [30] that has potential to improve the incorporation of cucumber resistance to disease.

The goals of this research were to isolate RGAs related to downy mildew resistance from line IL5211S based on degenerate primers designed from the conserved domains of the NBS-LRR of cloned R genes in plants, and to elucidate their character and genetic diversity. The transcriptional expression of putative CSRGA associated with downy mildew resistance after infection with *P. cubensis *and treatment molecules including MeJA, SA, ABA, and H_2_O_2 _was further investigated by qRT-PCR.

## Results

### Amplification and cloning of CSRGAs from IL5211S

Using a pair of degenerate primers, primer-R and primer-F, from previously published literature [31], a band of the predicted size (~500 bp) was observed by PCR amplification (not shown). The fragment was inserted into a pGEM-T Vector, and 35 recombinant clones were randomly selected and sequenced. Subsequently, a homology search was carried out for each of the 35 clone sequences using the BLASTX search in GenBank. Twenty-eight sequences were found to have a high sequence similarity with known R genes and RGAs from other plant species (Table 1). The remaining clones gave either sequence similarity with retrotransposon sequence or no match with R genes. These clones were not analyzed further in this paper.

**Table 1:** Best BLASTX hits of isolated CSRGAs with respect to RGAs from other plant species^a^.

Name	Accession number	Plant	Identity %	Similarity %	e- value
CSRGA17	AAQ73295	*Malus × domestica*	49	73	6e-36
CSRGA25	ABH06472	*Prunus avium*	52	71	1e-38
CSRGA23	AAU04762	*Cucumis melo*	87	95	7e-77
CSRGA20	ABK96821	*Cucurbita moschata*	83	93	2e-65

^a ^The region between the P-loop and GLPLA motifs (~170aa) of each cucumber RGA was used as a query in BLASTX searches.

### Sequence analysis

BLASTX searches found that the highest degree of identity between the 28 sequences and the known *N *resistance gene protein, RGAs from *C. melo*, reached 37.6 and 87%, respectively (See Additional File 1,). BLASTP searches of the deduced amino acid sequences of 28 sequences revealed the presence of the NR-ARC (nucleotide-binding and similarity to *Apaf-1*, R genes, and the *Ced-4*) domain. Further sequence analysis revealed the presence of no-stop codons or frameshift mutations in these sequences. Therefore, they were defined as CSRGAs.

In addition, sequence analysis found that amino acid substitutions occur in the NBS conserved domains in IL5211S. For example, the "V" residues of the conserved P-loop motif of most cloned CSRGAs was replaced by other residues ("S," "L," "M," "T", and "I"). The "L" residue of the GLPL motif of CSRGA9 and CSRGA15 was substituted for both "Fs." The amino acid substitutions of other conserved domains were also observed.

### Multiple alignment and phylogenetic analysis

**Figure 1:**
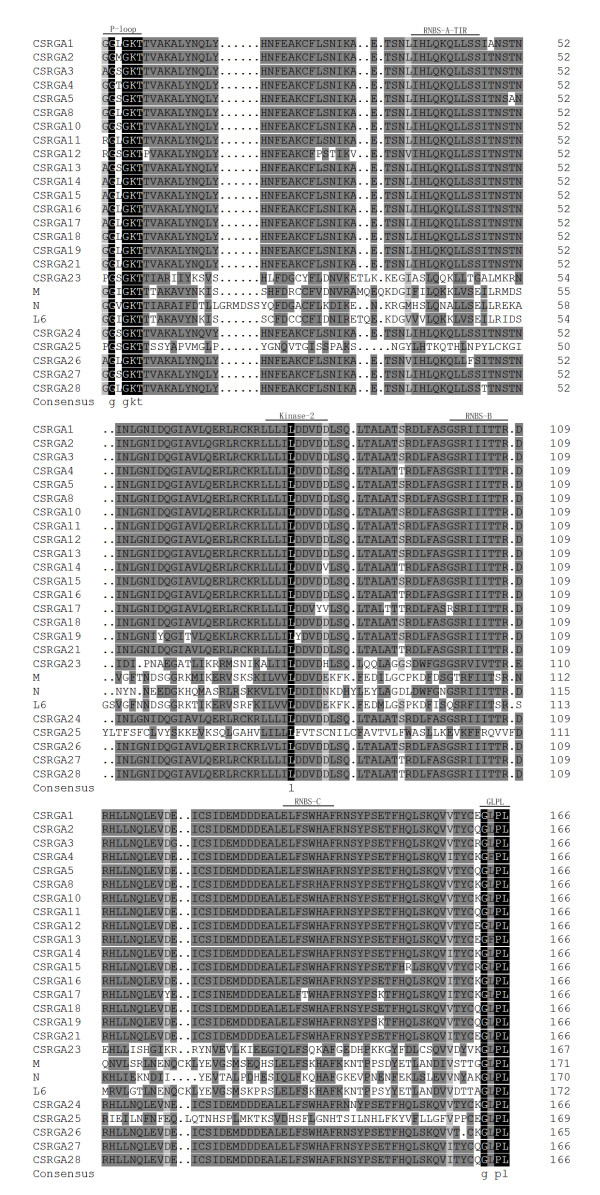
**Amino acid sequence between the P-loop and GLPL of TIR-CSRGAs with the NBS domains of known R genes, *N *(U15605), *L6 *(U27081), and *M *(U73916)**. Conserved domains are highlighted and indicated by an arrow. The alignment was constructed with DNAMAN 6.0 software.

**Figure 2:**
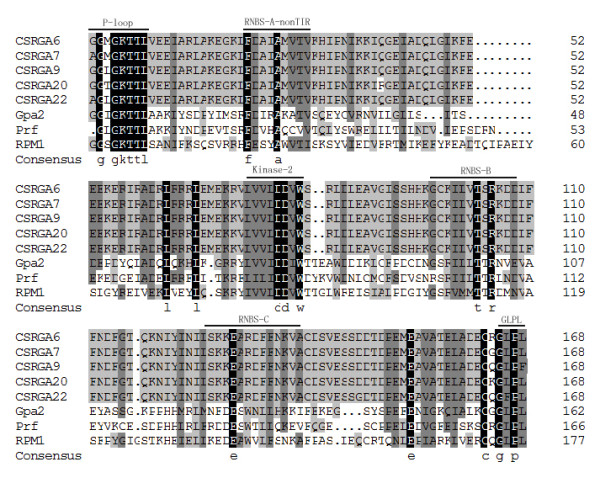
**Amino acid sequence between the P-loop and GLPL of nonTIR-NBS-LRR RGAs with the NBS domains of known R genes, Prf (U65391), Gpa2 (AF195939), and RPM1 (X87851)**. Conserved domains are highlighted and indicated by an arrow. The alignment was constructed with DNAMAN 6.0 software.

Multiple alignment between the 28 CSRGAs and known R genes including *N *(U15605), *L6 *(U27081), *M *(U73916), *Prf *(U65391), *Gpa2 *(AF195939), and *RPM1 *(X87851) revealed the presence of conserved domains such as P-loop, RNBS-A-TIR, RNBS-A-nonTIR, Kinase2, RNBS-B, and GLPL (Figures 1 and 2). Previous studies found that the P-loop, RNAS-B, and GLPL motifs were conserved in both TIR and non-TIR NBS-LRR resistance proteins, whereas the RNBS-A TIR (LQxQLLSxxL) and RNBS-A nonTIR (FDLxKxWVSVSDDF) motifs were found in the TIR and non-TIR NBS-LRR resistance proteins, respectively [8]. In addition, the amino acid residue at the end of Kinase2 is also a characteristic feature of TIR and non-TIR NBS-LRR resistance proteins. Tryptophan (W) corresponds to the non-TIR subclass of NBS-LRR resistance genes. In contrast, aspartic acid (D) corresponds to the TIR subclass. The method could be used to predict (95% accuracy) whether these resistance genes belong to the TIR or non-TIR subclass of NBS-LRR resistance genes [8]. Adopting these criteria, all CSRGAs cloned reported herein were divided into non-TIR and TIR subclasses. The former includes CSRGA6, CSRGA7, CSRGA9, CSRGA20, and CSRGA22. The remaining 23 CSRGAs belong to the TIR subclass.

**Figure 3:**
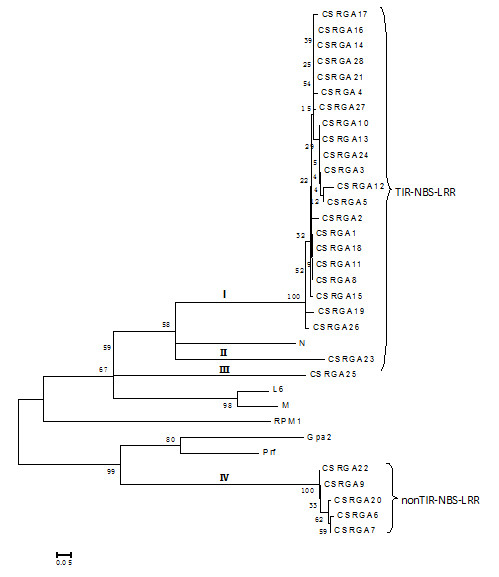
**Phylogenetic tree analysis based on the alignment of the deduced amino acid sequence of IL5211S RGAs with known R genes**. The tree was constructed using the neighbor-joining method provided in MEGA 4.0 software. The 28 RGA sequences were grouped into four subgroups: I and IV. I to III fall under the TIR-NBS-LRR-type RGA, whereas IV belonged to the non-TIR-NBS-LRR-type RGA. Bootstrap values (1,000 replicates) are given below the branches. The known R genes with the NBS domain used in this study were N (U15605), L6 (U27081), M (U73916), Prf (U65391), Gpa2 (AF195939), and RPM1 (X87851).

To explain the relationship of cloned CSRGAs and known R genes from other species, a phylogenic tree was constructed using MEGA4.0 software [32]. The genes were divided into two subgroups (Figure 3), which is consistent with our results. Within the TIR-NBS-LRR class, the CSRGAs are divided into three subclasses, designated as CSRGA I to CSRGA III (Figure 3). In the non-TIR-NBS-LRR class, the CSRGAs were clustered in one group, designated CSRGA IV.

Pairwise comparisons between the subclasses ranged from 33.8 to 51.2% at the nucleotide level and 5.7 to 26.8% at the amino acid level, suggesting a high degree of divergence and encoding a larger family of proteins with the NBS domain in IL5211S. Compared with known R genes, the sequence homology ranged from 23.9 to 41.7% at the nucleotide level and 4.9 to 37.6% at the amino acid level, respectively (See Additional File 1). The subclasses I and IV consisted of 21 and 5 members of CSRGAs, respectively. The remaining three subclasses included one member, reflecting a difference in abundance for these CSRGA subclasses in the cucumber genome (Figure 3).

### Analysis of the four groups of CSRGA gene expression

To detect the expression of the four groups of CSRGAs in IL5211S, one CSRGA representative of each group, namely, CSRGA17, CSRGA22, CSRGA23, and CSRGA25, was used in further analyses. Four primer pairs were employed to amplify from IL5211S using genomic DNA and complementary DNA (cDNA) as the template (See Additional File 2). The eight fragments obtained using these combinations were then cloned, and three clones of each were sequenced. Sequence comparisons indicated that no differences existed between the sequences amplified using genomic DNA and cDNA as template and original sequences, respectively. Thus, these results suggest that by using these four primers, the difference in expression levels observed could be attributable to the response to each of the CSRGA genes examined.

### Differential expressions of the four classes of CSRGAs in different organs of IL5211S

**Figure 4:**
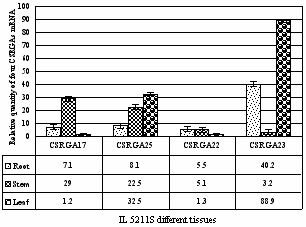
**Relative quantities of four CSRGAs with specific primers in different cucumber organs**. Each organ sample was individually assayed in triplicate.

To examine the expression levels of the four classes of CSRGAs in different plant organs from IL5211S, one CSRGA representative of each class was randomly chosen for expression analysis using RT-PCR. Results demonstrated that CSRGA23 was expressed in the leaves, stems, and roots at high levels, that CSRGA22 was expressed at relatively low levels, and the remaining two CSRGAs (CSRGA17 and CSRGA25) were expressed at intermediate levels. The expression level of the CSRGA23 gene in the leaves of IL5211S was also found to be significantly higher than that of the other three CSRGAs (Figure 4).

### Effects of introgression from *C. hystrix *on the four CSRGA transcription levels

**Figure 5:**
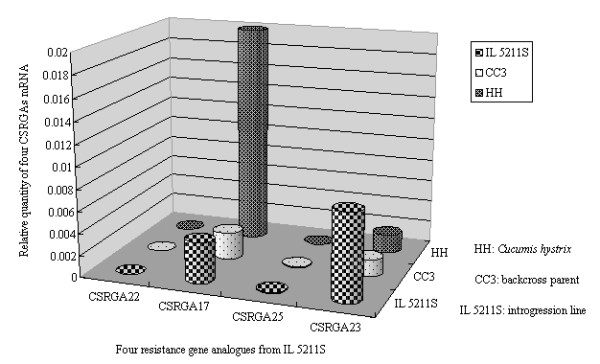
**Relative quantitative analysis of four CSRGAs within *Cucumis hystrix*, CC3, and IL5211S**.

To investigate whether alien DNA introgression from *C. hystrix *induced the expression of the four CSRGAs genes studied, qRT-PCR was used to analyze and compare their expression level in the wild relative, *C. hystrix*, the backcross-derived elite parent, "Beijingjietou," and line IL5211S. The results are presented in Figure 5. It indicates that CSRGA22 and CSRGA25 exhibited no remarkable changes in the transcription levels in IL5211S, *C. hystrix*, and CC3. In contrast, CSRGA17 and CSRGA23 showed differential expression. The expression level of CSRGA17 was about twice as high as observed in CC3. The level of CSRGA23 expression was four times higher than that of CC3. These results showed that introgression of *C. hystrix *induced expression of partial CSRGAs. Furthermore, the expression level of CSRGA23 was found to be higher than that of CSRGA17 in IL5211S, indicating that the role of CSRGA23 may be more important than CSRGA17 in enhancing downy mildew resistance in IL5211S.

### Induction of CSRGA23 expression in response to *P. cubensis *and defense signaling molecules

**Figure 6:**
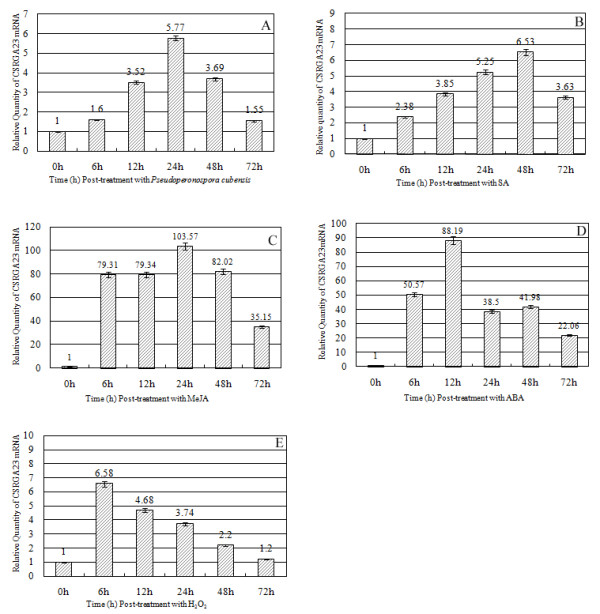
**Expression patterns of CSRGA23 mRNA using the qRT-PCR method in response to *Pseudoperonospora cubensis *and exogenous application of hormones and H_2_O_2_-treated seedlings**. (A) Seedlings were treated using *P. cubensis *inoculation (B) SA, (C) MeJA, (D) ABA, and (E) H_2_O_2_.

To understand the nature of CSRGA23 transcripts, the induction of CSRGA23 expression in response to *P. cubensis *and defense signaling molecules was examined by qRT-PCR (Figure 6A). Following infection by *P. cubensis*, the transcription of CSRGA23 was 1.6-fold higher at 6 h after inoculation. It increased to 3.5-fold at 12 h and peaked at 24 h (5.7-fold). Then the level of CSRGA23 expression decreased by 3.7-fold at 48 h and 1.5-fold at 72 h. This result suggests that CSRGA23 might be involved in the defense response to *P. cubensis*.

Previous studies have shown that SA and JA are signaling molecules involved in the activation of pathogenesis-related gene expression [33,34] and defense-related gene expression [35-37], respectively. Recent studies indicated that other hormones such as ABA are also involved in plant defense signaling pathways. In addition, H_2_O_2 _has been confirmed as a second messenger in activating defense gene expression [38] and is one of the earliest plant responses in incompatible interactions between pathogens and plants [39].

To evaluate whether expression of CSRGA23 was induced by various combinations of *P. cubense *infection with potential resistance inducing chemicals, the level of the CSRGA23 transcript in IL5211S leaves following exogenous application of SA, MeJA, ABA, and H_2_O_2 _was studied by qRT-PCR analysis. Following SA treatment, transcription of CSRGA23 occurred and reached 2.38-fold at 6 h after treatment. The expression of CSRGA23 increased to 5.25-fold at 24 h and peaked at 48 h. After this, the expression level decreased but was still at 3.63-fold at 72 h post-treatment, which is higher than the background (Figure 6B).

Exposure to MeJA, caused CSRGA23 transcription to increase rapidly to 79.31-fold at 6 h compared to initial exposure expression and this same level was maintained (79.34-fold) at 12 h, and peaked (103.57-fold) at 24 h. However, CSRGA transcription decreased abruptly after 24 h, at 82.02-fold, fell to 35.15-fold at 48 h and 72 h, respectively (Figure 6C).

Induction of CSRGA23 occurred rapidly after treatment with ABA. The expression level of CSRGA23 increased 50.57-fold at 6 h, peaked at 12 h (88.19-fold), then decreased to 38.5-fold at 24 h and had a minimal increase at 48 h compared to pre-exposure levels. After this, the expression level again decreased to 22.06-fold at 72 h (Figure 6D).

In response to H_2_O_2 _treatment, the expression level of CSRGA23 peaked at 6.58-fold higher 6 h, than that at 0 h. Then the expression of the gene decreased steadily: 4.68-fold at 12 h, 3.74-fold at 24 h, 2.2-fold at 48 h, and 1.2-fold at 72 h (Figure 6E).

Taken together, the strong induction of CSRGA23 by exogenous application of MeJA, SA, H_2_O_2_, ABA, and the observed *P. cubensis *infection, suggesting that CSRGA23 might be involved in defense responses via a signaling the pathway activated by these molecules.

## Discussion

Based on the successful interspecific hybridation between *C. hystrix *and *C. sativus*, high resistance to downy mildew cucumber IL was identified through cytogenetics and molecular genetics combined with *P. cubensis *inoculation [30]. To use the source of resistance to downy mildew fully, the determination of its defense mechanism in response to *P. cubensis *is necessary. In this paper, based on the conserved domains of plant NBS-LRR resistance genes, a pair of degenerate primers were used to amplify its homolog gene from IL5211S by PCR. Twenty-eight CSRGAs were successfully obtained.

Sequence alignment found that CSRGAs proteins have a highly conserved NR-ARC domain containing P-loop/Kinase-1a, Kinase-2, and Kinase-3a, and GLPL subdomains characteristic of most plant RGAs and R genes. In addition, according to the principle that the W residue is found in non-TIR proteins, and the D residue is only in TIR-containing proteins, 24 out of 28 (82%) of the CSRGAs examined in this work belonged to the TIR-NBS-LRR type. The remaining four CSRGAs belonged to the nonTIR-NBS-LRR type. This distribution is commonly found in *Arabidopsis *and *Tobacco *[40,41] and also supports the view that both TIR-NBS-LRR and that non-TIR-NBS-LRR *R*-genes occur in dicot species [9]. The phylogenetic tree separated CSRGAs into four subgroups belonging to two classes of the NBS-LRR sequence, which agrees with the results of our classification method.

To elucidate the expressed patterns of four CSRGAs in the different tissues from IL5211S, they were analyzed using qRT-PCR. Figure 4 shows that the expression levels of the four CSRGAs are different in the roots, stems, and leaves of IL5211S. The expression level of CSRGA23 was higher in leaves than in roots or stems. The expression levels of CSRGA17, CSRGA25, and CSRGA22 were highest in the stems, leaves, and roots, respectively.

The effects of introgression from *C. hystrix *on the four CSRGAs transcription levels were further investigated. The levels of the CSRGA26 and CSRGA25 transcript are almost the same among the distant relative *C. hystrix*, CC3, and IL5211S. The expression of CSRGA17 and CSRGA23 in IL5211S were induced by genetic factors originating from *C. hystrix*. The expression level of CSRGA23 is four-fold higher than that of CC3. These results show that introgression from *C. hystrix *caused a partial change in CSRGA expression. The enhanced expression level of CSRGA23 may be associated with downy mildew resistance in IL5211S.

Most plant resistance genes are transcriptionally regulated in response to pathogen attack. However, due to low expression levels, transcripts can be difficult to detect using gel blot analysis [42]. The transcription of the rice *Xa1 *resistance gene appears to increase following pathogen inoculation [43]. This indicates that the transcription of the R gene depends on the type of plant-pathogen interaction. In this study, the expression patterns of CSRGA23 were investigated to gain insight into its involvement in plant defense response (Figure 6A). Infection with *P. cubensis *significantly enhanced the expression level of CSRGA23, suggesting a correlation between activating this gene and resistance to *P. cubensis*. Thus, CSRGA23 might be involved in *P. cubensis-*induced defense response. Similar expression patterns have been observed for *Pib *and *Hs1^pro-1^*, a blast resistance gene in rice [44,45] and a nematode-resistance gene in sugar beet [46], respectively.

Previously, some studies have reported that signaling molecules not only function as a critical signal for downstream resistance events but also upregulate the expression of R genes. For example, in *Arabidopsis*, the study had shown that SA treatment induced the expression of *SSI4*, encoding a putative protein belonging to the TIR-NBS-LRR class of R proteins, and the closely related TIR-NBS-LRR genes *RPP1 *and *RPS4 *[47]. Treatment with SA also increases the transcription of *RPW8.1 *and *RPW8.2*, induces spontaneous HR-like lesions (SHL), and enhances resistance to powdery mildews [48]. In sugarcane and soybean, it has also been found that expression of the NBS-LRR class of resistance genes were induced by exogenous SA treatment and wounding [49-52]. In grapevines, of the *VvMLO *genes tested, the *VvMLO8 *and *VvMLO10 *genes showed over 50-fold higher transcript levels upon H_2_O_2 _treatment, while *VvMLO4*, *VvMLO6 *and *VvMLO10 *genes displayed the most marked response to SA treatment [53]. However, some researchers have also expressed opposite views. For example, expression of Ha-NTIR11g and *Hs1^pro-1 ^*(an RGA related to downy mildew infection in sunflower [54] and a nematode resistance gene in sugar beet [46]) were not induced by exogenous signaling molecules (i.e., ABA, H_2_O_2_). In this study, the *P. cubensis-*inducible CSRGA23 gene was activated not only by SA but also by other defense-signaling molecules (MeJA, ABA, and H_2_O_2_) (Figure 6B-6E), suggesting that these stimuli induce the expression of the CSRGA23 gene and that CSRGA23 may play a potential role in mediating cross-talk between defense-signaling pathways.

Several studies also report that SA and JA act antagonistically in defense reactions [55,56]. However, more recent studies indicate that they act synergistically [57]. In this paper, expression of CSRGA23 was induced by SA and MeJA, suggesting that the two stimuli have synergistic role in mediating defense responses in IL5211S. However, the CSRGA23 gene transcript-induced patterns were distinct. The CSRGA23 transcript-induced peak by SA occurred at 48 h and increased 6.53-fold over 0 h, whereas the transcript peak (103.5-fold increase over 0 h) induced by MeJA appeared at 24 h, suggesting that CSRGA23 might be mainly involved in the defense responses through signaling the pathway activated by these two molecules at different time points.

Taken together, these results suggest that the CSRGA23 gene is activated in IL5211S during infection by *P. cubensis*, and that it is induced by signal transduction pathways that mediated by SA, MeJA, ABA, and H_2_O_2_. Given the prominent role CSRGA23 proteins play in IL5211S defense responses, further examination of their function is necessary.

## Conclusions

In this study, 28 CSRGAs obtained by the homologue-sequence method provided the foundation for developing molecular markers and insight into downy mildew resistance formation. This contributes to our understanding of the organization of the NBS-LRR R gene in IL5211S, which may in turn result in future cloning of this "novel" downy mildew resistance gene.

## Methods

### Plant material, pathogen infection, and hormone treatments

**Figure 7:**
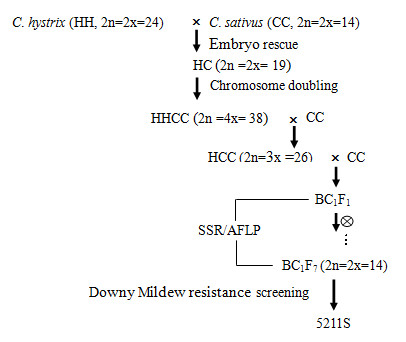
**Crossing procedure used to obtain downy mildew resistance introgression line, IL5211S, between *Cucumis hystrix *Chakr and cultivated cucumber (*C. sativus *L.) "Beijin jietou"**.

The procedure for obtaining IL5211S, an introgression line highly resistant to downy mildew from *C. hystrix *and *C. sativus*, was as follows (Figure 7). In the spring of 2008, seeds of line IL5211S were germinated and grown in growth chambers with 12 h light at 25°C and 12 h darkness at 18°C. Relative humidity was 65-75%. For the pathogen infection and hormone treatment, seedlings at the second true-leaf stage were treated with *P. cubensis *and signaling molecules containing SA (2 mM), MeJA (100 μM), and ABA (100 μM), respectively. For the H_2_O_2 _treatment, the same seedlings at the second true-leaves stage were sprayed with H_2_O_2 _(10 μM) in sterile water. Control plants were sprayed with sterile water only. The procedure for *P. cubensis *infection is as follows. The second true-leaf from each seedling was inoculated with a single drop (approximately 0.01 ml) of inoculum, containing 12 × 10^4 ^sporangia per milliliter. Then, the seedlings were placed inside plastic boxes and incubated at 20°C with approximately 100% relative humidity in the dark for 24 h. They were then placed in a chamber (24°C to 30°C) in a 16 h photoperiod.

### DNA isolation and primer design

The same seedlings in the second true-leaf stage from IL5211S were harvested, frozen immediately in liquid nitrogen, and stored at -80°C. Genomic DNA was isolated using a plant DNA extraction kit (Bioteke, China). A pair of degenerate primers were used to amplify the region between "P-loop" and "GLPL" of the plant R genes (See Additional File 3).

### PCR amplification and cloning

PCR reactions were performed in 25 μl mixtures containing 20 ng template DNA, 2 μl 10× PCR Buffer, 1.5 μl 25 mmol MgCl_2_, 2 μl dNTPs (2 mmol/L), 1 μl Primer-F (10 μmol/L), 1 μl Primer-R (10 μmol/L), 1 unit of *Taq *DNA (5 U/μl), and 15.3 μl ddH_2_O. PCR amplification was carried out in an MJ PTC-100 thermal cycler. The PCR reaction cycle program was as follows. The first step was denaturation at 94°C for 4 min, followed by 35 cycles of denaturation at 94°C for 30 s and annealing at 55°C for 30 s. The primer was extended at 72°C for 60 s, extending to 72°C for 5 min.

The DNA fragment (~500 bp) was collected from the electrophoresis gel and purified using a MinElute gel extraction kit (Qiagen). Then the resulting DNA was cloned into the pGEM-T easy vector (Promega, USA) and transformed into competent *Escherichia coli *JM 10^9 ^cells according to the instructions of the manufacturer. The cloned DNA fragment was sequenced by Bio-Asia Company (China).

### Sequence analysis, alignment, and phylogenetic tree analysis

After sequencing, the acquired DNA sequences were removed by vector with VecScreen in GenBank, compared in homologies with the GenBank database, and searched in the National Centre for Biotechnology Information (NCBI) GenBank using BLAST (Basic Local Alignment Search Tool) command http://www.ncbi.nlm.nih.gov. The percentage of amino acid identity between the predicted protein sequences was determined using DNAMAN6.0 computer software. The phylogenetic tree was constructed by the neighbor-joining (NJ) method using the NJ algorithm implemented in Molecular Evolutionary Genetics Analysis (MEGA) software version 4.0 [31]. Bootstrapping (1,000 replicates) was used to evaluate the degree of support for a particular grouping pattern in the phylogenetic tree.

### RNA extraction and cDNA preparation

Total RNA was isolated from the leaves of IL5211S using TRIzol reagent (Invitrogen) and treated with DNase I (Promega) to remove traces of genome DNA according to the manufacturer's instructions. The first strand cDNA synthesis was performed using olio (dT)_15 _primers (Promega) and 200 units of Moloney Murine Leukemia Virus (MMLV) reverse transcriptase (Promega) for 1 h at 42°C. Control reactions included a positive RT-PCR with actin-specific primers aside from a negative control with actin primers, but with RNA instead of cDNA as template to test for genomic DNA contamination. Amplicons were then electrophoresed on 1% agarose gel.

### CSRGA-specific primer design for expression analysis

Based on CSRGAs obtained from IL5211S, specific primer pairs were designed using Primer3.0 software. Four pairs of CSRGA-specific primer were obtained. The conditions for semi-RT-PCR amplification were standardized with genomic DNA from the respective taxon, and annealing temperatures were identified (See Additional File 2).

### Quantitative RT-PCR and data analysis

RT-PCR was conducted in a 25 μl mixture containing 12.5 μl 2× SYBRGreen PCR MasterMix (Applied Biosystems), 1 μl 10 pmoles of each primer, 1 μl template (15× diluted cDNA from leaf samples), and 9.5 μl sterile distilled water. The cucumber EF1a gene (EF446145) was used to normalize the sample [58]. The thermal conditions for RT-PCR were 95°C for 10 min (denaturation), followed by 40 cycles of 95°C for 15 seconds and 60°C for 1 min. All reactions were performed in triplicate. Quantification analysis was performed by the comparative C_T _method, which mathematically transforms the threshold cycle (C_T_) into the relative expression levels of genes (Perkin-Elmer User Bulletin). Data were analyzed using QPCR software (Rotor-Gene) and Microsoft Excel 2003.

## Abbreviations

QRT-PCR: quantitative real-time polymerase chain reaction; SA: salicylic acid; MEJA: methyljasmonic acid; ABA: abscisci acid; H_2_O_2_: hydrogen peroxide; JA: jasmonic acid; LZ: leucine zipper, NBS: nucleotide binding site; LRR: leucine-rich repeats; TM: transmembrane; PKS: serine/threonine protein kinases; RGA: resistance gene analog; NBS-LRR: nucleotide binding site and leucine-rich repeat.

## Authors' contributions

HJW participated in conceiving the paper, primer design, sequence analysis, and drafting the final manuscript. ZGZ participated in DNA extraction and PCR amplification. AAM participated in bioinformatics, and modified the final manuscript. CTQ participated in conceiving the study, and modified the final manuscript. JFC critically reviewed the manuscript and gave financial support to the study. All authors read and approved the final manuscript.

## Supplementary Material

Additional file 1 **Table S1**. The percentage of similarity among nucleotide acid sequences and amino acid sequences of cloned RGAs from IL5211S. The percentage of similarity among amino acid sequences and nucleotide acid sequences are given above and below the diagonal, respectively. Click here for file

Additional file 2 **Table S2**. RT-PCR amplification was used to determine the expression profiles of cloned RGA using the corresponding RGA-specific primers. Click here for file

Additional file 3 **Table S3**. Nucleotide acid sequences of degenerate primers used for amplifying and isolating of CSRGAs from IL5211S. Click here for file
