# Supplementary material for: Identification and characterization of potential NBS-encoding resistance genes and induction kinetics of a putative candidate gene associated with downy mildew resistance in Cucumis
Source: BMC Plant Biol. 2010 Aug 23;10:186. doi: 10.1186/1471-2229-10-186 (PMC2956536; doi:10.1186/1471-2229-10-186)
Supplement: Additional file 1 — Table S1. The percentage of similarity among nucleotide acid sequences and amino acid sequences of cloned RGAs from IL5211S. The percentage of similarity among amino acid sequences and nucleotide acid sequences are given above and below the diagonal, respectively. [file 1471-2229-10-186-S1.DOC]

Table S1: The percentage of similarity among nucleotide acid sequences and amino acid sequences of cloned RGAs from5 211S. The percentage of similarity among amino acid sequences and nucleotide acid sequences are given above and below the diagonal, respectively.

|  | CSRGA23 | CSRGA25 | CSRGA17 | Gpa2 | L6 | M | N | Prf | RPM1 |
| --- | --- | --- | --- | --- | --- | --- | --- | --- | --- |
| CSRGA20 | 6.3/38.3 | 6.2/33.8 | 6.3/41.3 | 12.9/23.9 | 4.9/27.1 | 6.8/29.0 | 8.0/24.3 | 6.3/24.3 | 6.6/32.2 |
| CSRGA23 |  | 5.7/39.8 | 26.8/51.2 | 7.2/30.5 | 15.7/29.1 | 15.2/29.3 | 37.6/30.9 | 8.3/32.2 | 18.1/41.7 |
| CSRGA25 |  |  | 9.9/35.5 | 9.0/30.0 | 9.8/26.7 | 9.8/26.7 | 6.2/29.2 | 8.1/24.8 | 5.4/24.0 |
| CSRGA17 |  |  |  | 11.7/25.5 | 26.2/31.2 | 25.1/29.6 | 28.5/36.2 | 10.8/39.7 | 9.6/31.1 |
| Gpa2 |  |  |  |  | 11.5/28.0 | 10.9/24.7 | 5.2/43.8 | 39.4/26.2 | 5.7/22.3 |
| L6 |  |  |  |  |  | 81.8/74.8 | 15.2/26.7 | 8.1/30.1 | 9.5/21.9 |
| M |  |  |  |  |  |  | 15.9/28.7 | 9.4/26.8 | 11.4/24.2 |
| N |  |  |  |  |  |  |  | 5.0/29.7 | 15.5/26.8 |
| Prf |  |  |  |  |  |  |  |  | 7.4/28.7 |
