# Supplementary material for: Identification and characterization of potential NBS-encoding resistance genes and induction kinetics of a putative candidate gene associated with downy mildew resistance in Cucumis
Source: BMC Plant Biol. 2010 Aug 23;10:186. doi: 10.1186/1471-2229-10-186 (PMC2956536; doi:10.1186/1471-2229-10-186)
Supplement: Additional file 2 — Table S2. RT-PCR amplification was used to determine the expression profiles of cloned RGA using the corresponding RGA-specific primers. [file 1471-2229-10-186-S2.DOC]

### Table S2: RT-PCR amplification was used to determine the expression profiles of cloned RGA using the corresponding RGA-specific primers.

| Clone name | Primer code | Sequence forward (5’ 3’) | Sequence reverse (5’ 3’) | Amplicon size(bp) | Temp (℃) |
| --- | --- | --- | --- | --- | --- |
| CSRGA17 | CS1 | TGGGAGGAATGGGGAAAAC | GCCCTCTACATTCATCTGC | 183 | 48 |
| CSRGA25 | CS2 | AATGTTTCCCTTCCACTATC | TCATCGTCATCCATTTCATC | 293 | 62 |
| CSRGA23 | CS3 | TCGGGGAAAACTACCATCG | CAGCCCTTGACATAATCTAC | 295 | 54 |
| CSRGA22 | CS4 | CGGGGAAAACTACAAGCAG | AACAAAGAAGCCCAGAACAG | 492 | 60 |
