# Supplementary material for: Identification and characterization of potential NBS-encoding resistance genes and induction kinetics of a putative candidate gene associated with downy mildew resistance in Cucumis
Source: BMC Plant Biol. 2010 Aug 23;10:186. doi: 10.1186/1471-2229-10-186 (PMC2956536; doi:10.1186/1471-2229-10-186)
Supplement: Additional file 3 — Table S3. Nucleotide acid sequences of degenerate primers used for amplifying and isolating of CSRGAs from IL5211S. [file 1471-2229-10-186-S3.DOC]

## Table S3: Nucleotide acid sequences of degenerate primers used for amplifying and isolating of CSRGAs from IL5211S.

| primer | Conserved motif | Primer sequence^a^ (5’to3’) | reference |
| --- | --- | --- | --- |
| prime-F (Forward) | P-loop (GGVGKTT) | TGSSRGGHWYRGGBAAAACTAC | Zhang et al. [**30**] |
| prime-R (Reverse) | HD (GLPLAL) | HRCWARAGGVARCCCTYBACA |

^a^The sequences are coded: M=A/C, R=A/G, W=A/T, S=G/C, Y=C/T, D=G/T, V=A/G/C, H=A/C/T, D=A/G/T, B=G/C/T, N=A/G/C/T
